# Evaluating awareness, knowledge and practice of healthcare professionals following implementation of a revised pregnancy prevention programme for isotretinoin in Ireland: A multi‐stakeholder cross‐sectional study

**DOI:** 10.1002/pds.5538

**Published:** 2022-10-05

**Authors:** John E. Hughes, Niamh Buckley, Yvonne Looney, Gráinne Kirwan, Maeve Mullooly, Kathleen E. Bennett

**Affiliations:** ^1^ School of Population Health RCSI University of Medicine and Health Sciences Dublin 2 Ireland; ^2^ Health Products Regulatory Authority Kevin O'Malley House, Earlsfort Terrace Dublin 2 Ireland; ^3^ Data Science Centre, School of Population Health RCSI University of Medicine and Health Sciences Dublin 2 Ireland

**Keywords:** isotretinoin, patient safety, pharmacoepidemiology, pharmacovigilance, pregnancy prevention program, teratogenicity

## Abstract

**Purpose:**

In 2018, following an EU‐wide safety review, a revised pregnancy prevention programme (PPP) was introduced for isotretinoin (Roaccutane®). This study aimed to examine awareness, knowledge, and experience implementing the revised isotretinoin PPP in clinical practice across three healthcare professional (HCP) groups in Ireland.

**Methods:**

A cross‐sectional study using anonymous online surveys among general practitioners (GPs), community pharmacists, and specialist consultants was undertaken. Descriptive analyses are presented.

**Results:**

Across all HCP groups there was high (≥87%) awareness that oral isotretinoin is contraindicated in women of childbearing potential (WCBP) unless the conditions of the PPP are fulfilled, but varying awareness among GPs (54.9%) and community pharmacists (45.9%) that exposure during pregnancy can cause both severe fetal malformations and spontaneous abortions. Implementation of the PPP in clinical practice varied across HCP groups. When initiating isotretinoin in WCBP, 66.7% of specialists and 40.8% of GPs indicated they had considered alternative treatment options, and 71.4% of specialists and 31.6% of GPs reported they first requested a pregnancy test. There was limited provision of the patient reminder card to WCBP, where 26.1% of community pharmacists provide this at each dispensing, while 47.6% of specialists and 11.8% of GPs ensured WCBP had a copy of the card when initiating treatment. Across all HCP groups, there was high (≥81.6%) awareness of the need for urgent consultation and immediate cessation of isotretinoin in the event of an unplanned or suspected pregnancy.

**Conclusions:**

Reinforcement of the provision and utilisation of the isotretinoin patient reminder card may be required, and further targeted education on specific elements of the PPP should be considered for GPs and community pharmacists.

**Key Points**

1. Awareness amongst responding specialist prescribers on all aspects of the PPP was high.
2. Amongst healthcare professionals for whom survey questions were applicable, most implemented all aspects of the PPP in clinical practice.
3. Further education on specific elements of the revised PPP may be of value for general practitioners and community pharmacists.
4. Amongst all healthcare professionals, reinforcement of the provision and utilisation of the isotretinoin patient reminder card may also be beneficial.

**Plain Language Summary**

Isotretinoin (Roaccutane®) is a medication used for severe acne. Exposure to isotretinoin during pregnancy, even for short time periods, is associated with a high risk of severe and life‐threatening pregnancy outcomes. To minimize these risks, a pregnancy prevention program (PPP) has been in place since 1988, and was updated in 2018 by the European Medicines Agency. Our study used anonymous online surveys to examine awareness, knowledge, and experience implementing the revised isotretinoin PPP in clinical practice in Ireland among general practitioners (GPs), community pharmacists, and specialist consultants. Our results reveal high (≥87%) awareness that oral isotretinoin should not be used in women of childbearing potential (WCBP) unless the conditions of the PPP are fulfilled. Some variation was evident regarding implementation of the PPP in clinical practice; there was also limited provision of the patient reminder card to WCBP. Across all healthcare professional groups, our results show high (≥82%) awareness of the need for urgent consultation and immediate cessation of isotretinoin in the event of an unplanned or suspected pregnancy. Collectively, our findings show that awareness of the PPP was high; however, further education and reinforcement of specific elements of the revised PPP may be beneficial.

## 1 INTRODUCTION

Isotretinoin (Roaccutane®), a vitamin A derivative, is an effective treatment for severe acne resistant to adequate courses of standard therapy with systemic anti‐bacterials and topical therapy.^1^ Like all vitamin A derivatives, isotretinoin is highly teratogenic and exposure during pregnancy, even for short time periods, is associated with a high risk of severe and life‐threatening congenital malformations and an increased risk of spontaneous abortion.^2^, ^3^ In utero exposure to oral retinoids is characterised by retinoic acid embryopathy, which is associated with malformations of central nervous system, craniofacial, cardiac and thymic structures,^4^, ^5^, ^6^ and is estimated to occur in approximately 26% of exposed patients.^6^

A pregnancy prevention programme (PPP) for Roaccutane® was first introduced in 1988 by the marketing authorisation holder (MAH) to minimise the risk of embryopathy.^7^ In 2003, with the availability of generic formulations of isotretinoin, divergences in the product information were evident across the EU, and a European review was initiated to ensure harmonisation across Member States. An EU PPP was agreed that was applicable to all oral isotretinoin‐containing medicinal products.^8^ In addition, the indication for isotretinoin was restricted to second‐line use. Previous studies which examined the implementation of and adherence to these earlier isotretinoin PPPs, largely report poor HCP uptake and compliance in practice.^7^, ^9^, ^10^, ^11^ A 2009 study examined the implementation of the harmonised EU‐PPP for isotretinoin in Member States and found that although 95% of responding countries (*n* = 22) had implemented the PPP, 143 isotretinoin‐exposed pregnancies were reported in 16/22 responding Member States since its implementation in 2003.^9^ In July 2016, following concerns relating to the implementation and effectiveness of the PPP for isotretinoin and other retinoid‐containing medicinal products, the European Medicines Agency's (EMA) Pharmacovigilance Risk Assessment Committee (PRAC) initiated a review of these products to evaluate the measures in place for pregnancy prevention. Following this, in March 2018, the PRAC recommended strengthening the recommendations for pregnancy prevention during treatment with certain oral retinoids, including isotretinoin.^12^ The educational materials for Roaccutane in place prior to the 2018 referral were: ‘Physician's guide to prescribing Isotretinoin (Roaccutane®)’, ‘Pharmacist's guide to dispensing Roaccutane® (isotretinoin)’, ‘Roaccutane Pregnancy Prevention Programme Checklist for Prescribing to Female Patients’, ‘Patient information brochure’, ‘Contraception Booklet’, and an ‘Acknowledgement form for female patients’. As an outcome of the 2018 referral, the PPP was harmonised and supported by a streamlined set of materials consisting of a ‘Physician Checklist/Acknowledgement Form for Prescribing isotretinoin to Female Patients’ (for patients and prescribers to go through and confirm that appropriate advice has been given and understood), a ‘Pharmacist Checklist/Guidance for dispensing isotretinoin’ and a ‘patient reminder card’ which states that the medicine must not be used during pregnancy, and which also includes information about pregnancy testing and the need to use effective contraception.

In Ireland, the Health Products Regulatory Authority (HPRA) liaised with the MAH responsible for marketing oral isotretinoin‐containing medicinal products nationally to ensure healthcare professionals (HCPs) were informed of the PRAC recommendations in order to facilitate their implementation in clinical practice. A number of communications were issued to prescribers and pharmacists and revised educational materials were distributed to these groups by the MAH in December 2018. The present study aimed to examine awareness, knowledge, and experience implementing the revised isotretinoin PPP in clinical practice across three HCP groups (general practitioners (GPs), community pharmacists, and specialists in dermatology and obstetrics and gynecology) in Ireland.

## 2 METHODS

The Strengthening the Reporting of Observational Studies in Epidemiology (STROBE) guidelines were adhered to in the reporting of this study.^13^

### 2.1 Study design

A cross‐sectional anonymous survey study was conducted that aimed to examine the effectiveness of risk minimisation measures in preventing harm from teratogenic medicines within an Irish healthcare setting. Further information on the study design and methods is previously described.^14^ Briefly, the overarching survey design was adapted for the 2018 isotretinoin PPP, which outlines specific actions for prescribers and pharmacists to minimise the risk of exposure to isotretinoin during pregnancy. The implementation of the PPP is supported by regulatory‐approved educational materials to facilitate implementation in clinical practice. These consist of a physician checklist/acknowledgement form^15^ for those prescribing isotretinoin to female patients, a pharmacist checklist^16^ for those dispensing isotretinoin to all patients, and a patient reminder card.^17^ In order to minimise the teratogenic risk of isotretinoin, information about the risk and the pregnancy prevention measures must be provided by the physician to female patients, supported by completion of the physician checklist and provision of the patient reminder card.^15^, ^17^ The pharmacist must also review and complete the relevant checklist before dispensing isotretinoin to all patients, using the patient reminder card to support patient counselling.^16^, ^17^ Therefore, for the purpose of this current study, representative samples of relevant prescribers (GPs and specialist consultants/higher specialist trainees [HSTs] in dermatology/obstetrics and gynecology) and pharmacists were included. While obstetricians and gynecologists are less likely to prescribe isotretinoin, they may be involved in the care of women of childbearing potential (WCBP) who use retinoids, and they were recipients of the 2018 “Direct Healthcare Professional Communication” (DHPC) and educational materials. The participating HCPs were identified via their respective professional representative or regulatory body as outlined previously.^14^

### 2.2 Survey development and distribution

Three isotretinoin surveys, adapted to each of the three HCP groups (GPs, specialist consultant/HSTs and pharmacists), were devised in conjunction with the HPRA (Electronic Supplementary file 1). The surveys focused on ‘process’ indicators for effectiveness and evaluated three areas relating to the isotretinoin PPP: (1) awareness and knowledge of key elements of the revised PPP and the risks associated with use of isotretinoin during pregnancy; (2) awareness and use of regulatory‐approved educational materials to support implementation into clinical practice; and (3) experience implementing the PPP in the HCP's clinical practice. Demographic information (i.e., age, gender, number of years in practice, and time since qualification) was also gathered. The surveys were piloted in a sample of HCPs (*n* = 4) prior to distribution and minor changes were made to some questions based on feedback received, to improve overall clarity.

A random sample of GPs (*n* = 1546) and specialist consultants (obstetricians/gynecologists (*n* = 156) and dermatologists (*n* = 72)) were contacted by their respective professional representative bodies between June and August 2019. A random sample of pharmacists (*n* = 2081), including community and hospital pharmacists, were also invited to complete the survey between June and August 2019. HCPs were provided with the survey link (Survey Monkey®) via an email invitation to participate. A follow‐up reminder to complete the survey was sent 2–4 weeks following the initial distribution.

### 2.3 Statistical analysis

Descriptive analyses are presented. The response rate for each HCP group was calculated as the number who completed the survey out of the total contacted via email. The percentage responses are calculated using denominators of those who responded to each question. As some questions were contingent upon others (e.g., Q2 is contingent on Q1), the percentage for such questions is calculated using the numerator of the contingent question. For the purpose of this study, only responses from pharmacists working in community practice (48.7% of pharmacist respondents) were included as they are more likely to dispense and advise on isotretinoin regularly. Since the pharmacist survey examined pharmacy practice relating to both men and women prescribed isotretinoin, the results presented for community pharmacists' experience in practice also include results relating to their experience implementing the PPP with male patients. Understanding and knowledge type survey questions were assessed by the number and percentage of HCP responders who passed the survey (i.e., ≥80% correct responses). Data where *n* < 5 are not presented. To assess the generalisability of the responding HCP population, age and gender distributions between respondents and the wider HCP population from which they were sampled were compared descriptively. This was only possible for GPs and community pharmacists, as specialist respondents were not asked about their area of specialisation.

## 3 RESULTS

### 3.1 Overview of HCP population response

Overall response rates were low with 6.3% of GPs (98/1546), 11.0% of pharmacists (228/2081), and 11.4% of specialists (dermatologist/obstetrics and gynecology; 26/228) responding. For this study, only responses from pharmacists working in community practice (*n* = 111, 5.3% response rate) are reported. Therefore, data from 235 HCP respondents (*n* = 98 GPs, *n* = 111 community pharmacists, and *n* = 26 specialists) were included in the analysis. A greater proportion of all responders were female (GPs: 66.7%; community pharmacists: 68.5% and specialists: 81.0%), and the GP and community pharmacist demographic data (by age of respondents) showed a similar distribution to Irish HCP general population data (Supplementary Tables S1–S3).

### 3.2 Awareness and knowledge of the isotretinoin PPP

**FIGURE 1:**
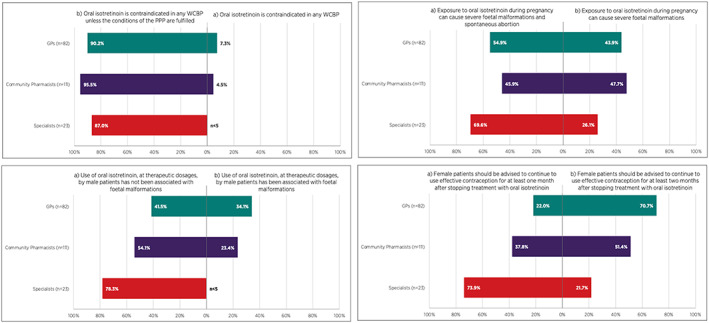
Figure showing responses to survey Q3 where HCPs were asked to indicate which statement in the following pairs is most accurate (correct answer option presented on left hand side of each graph). WCBP, woman of childbearing potential, PPP, pregnancy prevention programme. Where the percentages do not sum to 100% within each HCP group, this reflects the proportion of HCPs who responded “not sure”

Overall, 41.8% of GPs, 58.6% of community pharmacists and 46.2% of specialists indicated that they had received information relating to the revised PPP for isotretinoin. The most common source of this information cited by GPs and pharmacists was the “Dear Dr/Pharmacist letter” (DHPC from the MAH, approved by the HPRA), while educational materials was the source cited by 50% of responding specialists (Supplementary Table S4). Regarding isotretinoin's conditions for use, 90.2% of GPs, 95.5% of community pharmacists, and 87.0% of specialists correctly identified oral isotretinoin to be contraindicated in any WCBP unless the conditions of the PPP are fulfilled (Figure 1).

Awareness of the teratogenic risks associated with exposure to oral isotretinoin varied across the HCP groups (Figure 1). Among responding GPs and community pharmacists, 43.9% and 47.7% respectively, did not correctly identify that exposure to oral isotretinoin during pregnancy can cause both severe fetal malformations and spontaneous abortions, while 69.6% of specialists correctly identified both of these risks. Knowledge of the need for continued use of effective contraception upon cessation of treatment with oral isotretinoin also varied. Among responding HCPs, 70.7% of GPs and 51.4% of community pharmacists overestimated the timeframe for which female patients should be advised to continue using effective contraception, whereas 73.9% of specialists correctly identified that effective contraception should be continued for at least 1 month following cessation of oral isotretinoin treatment (Figure 1).

### 3.3 Awareness and use of isotretinoin PPP educational materials

**FIGURE 2:**
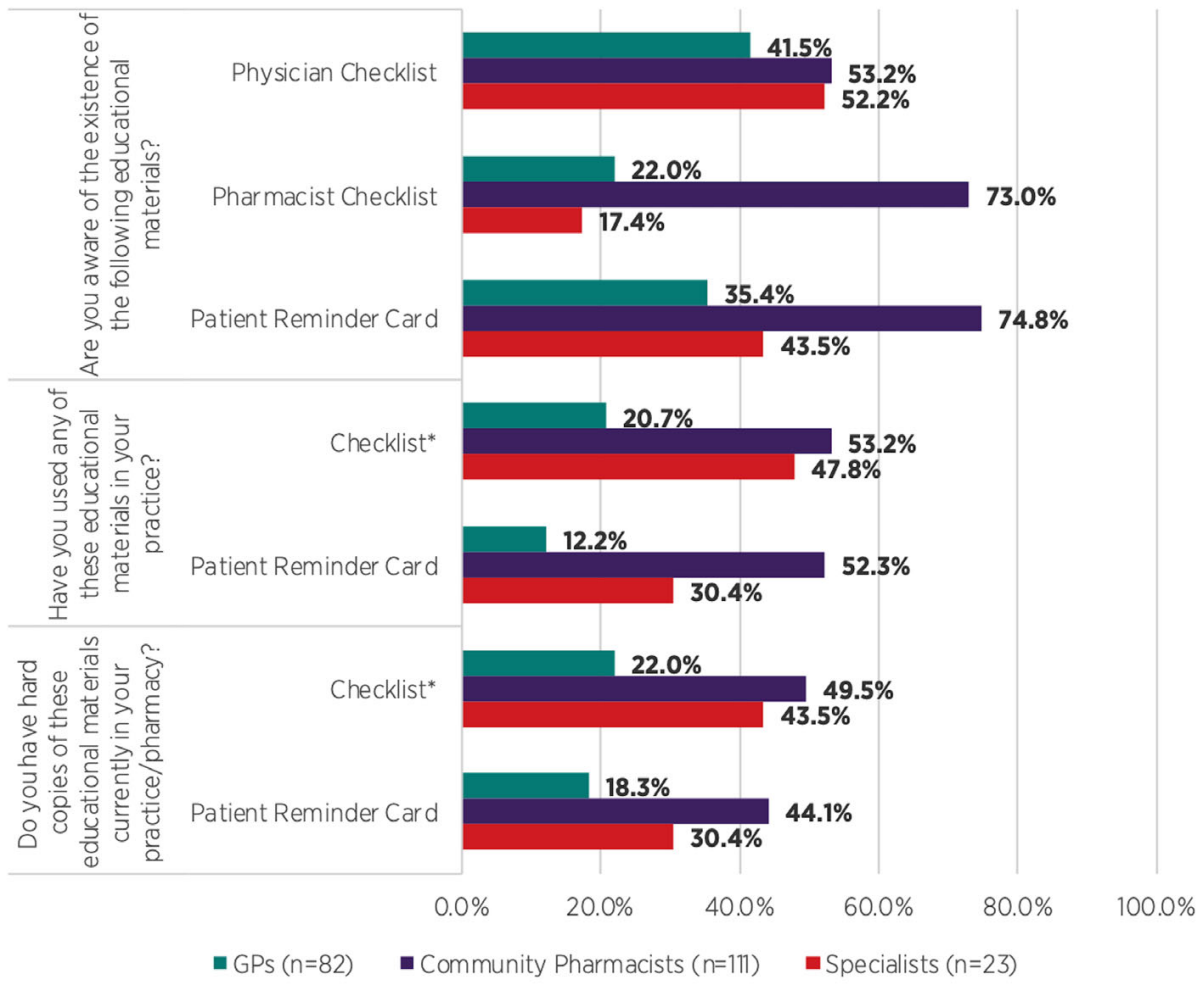
Awareness and use of the isotretinoin PPP educational materials in GP & specialist clinical practice and pharmacy practice. *Only the relevant HCP checklist is applicable (i.e., Physician Checklist for GP & specialist respondents and Pharmacist Checklist for community pharmacist respondents)

Awareness and use of educational materials to support implementation of the revised PPP varied across HCP respondents (Figure 2). Awareness of educational materials was higher among community pharmacists and comparatively lower amongst specialists and GPs. Correspondingly, use of the educational materials in practice was higher among responding community pharmacists, while fewer responding GPs and specialists indicated they had used these educational materials in their practice (Figure 2). However, 28.0% (*n* = 23) of GP respondents and 21.7% (*n* = 5) of specialist respondents indicated that use of the physician checklist and patient reminder card was not applicable to their practice.

### 3.4 Experience of GPs and specialists implementing the revised isotretinoin PPP into clinical practice

The reported isotretinoin prescribing patterns among responding GPs and specialists are shown in Table 1. Most GPs indicated that they had not initiated (82.9%), but had renewed (53.7%), and 56.5% of responding specialists reported they had initiated and renewed a prescription for isotretinoin in the past 12 months. However, 51.2% of responding GPs and 43.5% of responding specialists reported never prescribing isotretinoin (Table 1).

**TABLE 1:** Clinical practice following the implementation of the PPP in HCP's that prescribe medication (*n* [%])

		GPs (*n* = 82)	Specialists (*n* = 23)
When was the last time you initiated a new prescription for Roaccutane®?	In the past 12 months	14 (17.1%)	13 (56.5%)
	Did not initiate a new prescription for Roaccutane® in the past 12 months	68 (82.9%)	10 (43.5%)
When was the last time you renewed a prescription for Roaccutane®?	In the past 12 months	44 (53.7%)	13 (56.5%)
	Did not renew a prescription for Roaccutane® in the past 12 months	38 (46.3%)	10 (43.5%)
On average, how frequently do you prescribe Roaccutane®?	Occasionally to frequently (about once per month to greater than once per week)	13 (15.9%)	13 (56.5%)
	Rarely to Very rarely (about once every 3–6 months to about once every 12 months)	27 (32.9%)	0 (0.0%)
	Never	42 (51.2%)	10 (43.5%)

			GPs (*n* = 76)	Specialists (*n* = 21)
Which, if any, of the following have you implemented in your clinical practice with WCBP when initiating Roaccutane®?	I considered alternative treatment options before initiating Roaccutane®	Yes	31 (40.8%)	14 (66.7%)
		No	0 (0.0%)	*n* < 5
		Not applicable	45 (59.2%)	6 (28.6%)
	I initiated my patient on effective contraception (if not already using contraception)	Yes	33 (43.4%)	10 (47.6%)
		No	0 (0.0%)	*n* < 5
		Not applicable	43 (56.6%)	7 (33.3%)
	I referred my patient to her GP/family planning clinic for contraceptive advice (if not already using contraception)	Yes	—	13 (61.9%)
		No	—	*n* < 5
		Not applicable	—	6 (28.6%)
	I ensured my patient understood the need to comply with contraception for 1 month before starting treatment, throughout treatment and for 1 month after the end of treatment	Yes	35 (46.1%)	15 (71.4%)
		No	0 (0.0%)	0 (0.0%)
		Not applicable	41 (53.9%)	6 (28.6%)
	I requested my patient to have a pregnancy test before starting treatment with Roaccutane®	Yes	24 (31.6%)	15 (71.4%)
		No	6 (7.9%)	0 (0.0%)
		Not applicable	46 (60.5%)	6 (28.6%)
	I ensured my patient understood the teratogenic risks associated with use of Roaccutane**®** in pregnancy	Yes	33 (43.4%)	16 (76.2%)
		No	0 (0.0%)	0 (0.0%)
		Not applicable	43 (56.6%)	5 (23.8%)
	I ensured my patient had a copy of the Patient Reminder Card	Yes	9 (11.8%)	10 (47.6%)
		No	16 (21.1%)	5 (23.8%)
		Not applicable	51 (67.1%)	6 (28.6%)

			GPs (*n* = 76)	
Which, if any, of the following have you implemented in your clinical practice with WCBP when renewing a prescription for Roaccutane®?	I limit my prescriptions to 30 days to support regular follow‐up	Yes	30 (39.5%)	—
		No	15 (19.7%)	—
		Not applicable	31 (40.8%)	—
	When indicated, I request my patient to have a pregnancy test	Yes	39 (51.3%)	—
		No	5 (6.6%)	—
		Not applicable	31 (40.8%)	—

			GPs (*n* = 76)	Specialists (*n* = 21)
In the event of an unplanned or suspected pregnancy in your patient currently being prescribed Roaccutane®, which, if any, of the following would you implement in your clinical practice?	I would advise my patient to consult me urgently	Yes	66 (86.8%)	21 (100.0%)
		No	*n* < 5	0 (0.0%)
		Not applicable	9 (11.8%)	0 (0.0%)
	I would advise my patient to consult with her GP urgently	Yes	—	19 (90.5%)
		No	—	*n* < 5
		Not applicable		0 (0.0%)
	I would advise my patient to stop taking Roaccutane® immediately	Yes	62 (81.6%)	21 (100.0%)
		No	*n* < 5	0 (0.0%)
		Not applicable	10 (13.2%)	0 (0.0%)
	I would reduce my patients' Roaccutane**®** dose	Yes	*n* < 5	*n* < 5
		No	37 (48.7%)	19 (90.5%)
		Not applicable	26 (34.2%)	0 (0.0%)
At what stage(s) in the patient journey do you review teratogenic risks of Roaccutane® with female patients?		On initiation of Roaccutane**®**	40 (52.6%)	12 (57.1%)
		If patient is planning a pregnancy	13 (17.1%)	6 (28.6%)
		Patient is pregnant	*n* < 5	*n* < 5
		Annually	0 (0.0%)	*n* < 5
		At every consultation	35 (46.1%)	16 (76.2%)
		Do not review risks with patients	*n* < 5	0 (0.0%)
		Not applicable	22 (28.9%)	*n* < 5
Have there been clinical scenarios in your practice where you have adapted the PPP for Roaccutane® to an individual patient's circumstances?		Yes	*n* < 5	10 (47.6%)
		No	38 (50.0%)	5 (23.8%)
		Not applicable	36 (47.4%)	6 (28.6%)

*Note*: Data presented as *n* (%), unless otherwise stated. The dashed line indicates that this question/answer was not asked/included in the respective HCP survey.

Regarding their experience implementing the PPP in clinical practice when initiating isotretinoin in WCBP, two‐thirds (66.7%) of specialists and 40.8% of GPs indicated that they had considered alternative treatment options; 71.4% of specialists and 31.6% of GPs indicated that they requested their patient to have a pregnancy test before initiating isotretinoin; and less than half of all responding specialists (47.6%) and 11.8% of GPs indicated they ensured their patient had a copy of the patient reminder card. Approximately half (51.3%) of responding GPs indicated that they request their patient to have a pregnancy test when renewing a prescription for isotretinoin (Table 1). However, more than half of all responding GPs indicated that these questions were “not applicable” to their clinical practice (Table 1). Other key elements of the isotretinoin PPP relevant to GP and specialist practice are presented in Table 1.

Among the responding GPs and specialists who indicated that they never prescribe or frequently/occasionally prescribe Roaccutane®, we conducted subgroup analyses to examine their awareness/knowledge/experience of the revised isotretinoin PPP (see Supplementary Table S5). The responding specialists who indicated that they frequently/occasionally prescribe Roaccutane® had greater awareness/knowledge of the revised isotretinoin PPP compared to that of the responding GPs. Across both HCP subgroups, there was limited awareness of and experience using the patient reminder card (see Supplementary Table S5).

### 3.5 Experience of community pharmacists implementing the revised PPP into practice

Community pharmacists' experience implementing the revised PPP into practice for WCBP, and where applicable for men, is outlined in detail in Table 2. Overall, 82.9% indicated that they had dispensed isotretinoin in the past 12 months. Further, when dispensing to WCBP currently prescribed isotretinoin, 81.1% ensured, where possible, that the prescription was limited to a 30 day supply and 73.0% reported that they ensured it was dispensed within a maximum of 7 days of the prescription date. With regard to counselling WCBP on the teratogenic risks, 59.5% of community pharmacists indicated that they do this only for new patients, while 27.0% reported that they do this at each dispensing. Similarly, in relation to provision of the patient reminder card (and related counselling) to WCBP, 45.9% of responding community pharmacists indicated that they only do this for new patients, whereas 26.1% reported they do so at each dispensing. The proportion of responding community pharmacists who indicated that these questions were “not applicable” to their clinical practice ranged from 4.5% to 57.7% (Table 2). Other key elements of the isotretinoin PPP relevant to pharmacy practice are presented in Table 2.

**TABLE 2:** Clinical practice following the implementation of the PPP in HCP's that dispense medication (*n* [%])

		Community pharmacists (*n* = 111)
Have you dispensed Roaccutane® in the past 12 months?	Yes	92 (82.9%)
	No	17 (15.3%)
	Not applicable	*n* < 5
When dispensing Roaccutane®, which, if any, of the following have you implemented in your pharmacy practice with WCBP currently being prescribed Roaccutane®?	I ensured Roaccutane® was only dispensed within a maximum of 7 days of the prescription	
	Yes	81 (73.0%)
	No	11 (9.9%)
	Not applicable	19 (17.1%)
	Where possible, I ensured the prescription was limited to a 30 day supply	
	Yes	90 (81.1%)
	No	*n* < 5
	Not applicable	16 (14.4%)
	I referred my patient to her GP as she was not using effective contraception	
	Yes	17 (15.3%)
	No	28 (25.2%)
	Not applicable	64 (57.7%)
When dispensing Roaccutane®, which, if any, of the following have you implemented in your pharmacy practice with men and WCBP currently being prescribed Roaccutane®?^a^	I advised my patient never to share their Roaccutane® with another person	
	Yes	Men: 55 (49.5%) WCBP: 65 (58.6%)
	Not applicable	41 (36.9%)
	I advised my patient to return any unused capsules to me at the end of the month	
	Yes	Men: 43 (38.7%) WCBP: 50 (45.0%)
	Not applicable	47 (42.3%)
	I advised my patient not to donate blood during Roaccutane® therapy and for 1 month after discontinuation	
	Yes	Men: 33 (29.7%) WCBP: 42 (37.8%)
	Not applicable	48 (43.2%)
How often do you provide the patient card to your patient and counsel her on its contents?	Only for new patients	51 (45.9%)
	At each dispensing	29 (26.1%)
	Only if the patient initiates discussion	*n* < 5
	Never	19 (17.1%)
	Not applicable	9 (8.1%)
How often do you counsel the patient on the teratogenic risks associated with use of Roaccutane® in pregnancy?	Only for new patients	66 (59.5%)
	At each dispensing	30 (27.0%)
	Only if the patient initiates discussion	5 (4.5%)
	Never	5 (4.5%)
	Not applicable	5 (4.5%)
How often do you reinforce the need for effective contraception?	Only for new patients	48 (43.2%)
	At each dispensing	43 (38.7%)
	Only if the patient initiates discussion	9 (8.1%)
	Never	*n* < 5
	Not applicable	6 (5.4%)
When dispensing Roaccutane® to a male patient, how often do you provide the patient reminder card and counsel him on the relevant aspects of its contents?	Only for new patients	59 (53.2%)
	At each dispensing	14 (12.6%)
	Only if the patient initiates discussion	10 (9.0%)
	Never	19 (17.1%)
	Not applicable	9 (8.1%)
When dispensing Roaccutane® and broken bulk dispensing cannot be avoided, which, if any, of the following do you provide to the patient?^a^	Roaccutane® package leaflet/patient information leaflet (only for WCBP)	30 (27.0%)
	Roaccutane® package leaflet/patient information leaflet (for men and WCBP)	84 (75.7%)
	Roaccutane® patient card (only for WCBP)	20 (18.0%)
	Roaccutane® patient card (for men and WCBP)	39 (35.1%)
	None of the above	*n* < 5
	Not applicable	8 (7.2%)
In the case of an unplanned or suspected pregnancy in a woman currently being prescribed Roaccutane®, which, if any, of the following actions would you take?^a^	I would advise my patient to contact her prescribing doctor immediately	111 (100.0%)
	I would advise my patient to stop taking Roaccutane® immediately	110 (99.1%)
	I would not dispense Roaccutane® to my patient	110 (99.1%)
	I would counsel my patient on the teratogenic risks associated with use of Roaccutane® in pregnancy	105 (94.6%)

*Note*: Data presented as *n* (%), unless otherwise stated.

^a^ Pharmacists may select more than one option, if applicable.

## 4 DISCUSSION

### 4.1 Main findings

This study examined HCP awareness, knowledge, and experience implementing the revised isotretinoin PPP in clinical practice in Ireland. The survey findings show wide variability in awareness, with approximately 40%–60% of responding HCPs indicating that they had received information relating to the revised isotretinoin PPP. Most (over 87%) HCPs correctly identified that isotretinoin is contraindicated in WCBP unless the conditions of the PPP are fulfilled. However, compared to specialist respondents, when asked about the nature of the teratogenic risk, there was limited awareness amongst responding GPs and community pharmacists that exposure to oral isotretinoin during pregnancy was associated with a risk of both fetal malformations and spontaneous abortions. Survey findings highlight that while the majority of specialists indicated that they had implemented all elements of the PPP when initiating isotretinoin in WCBP, compliance with implementation of the revised PPP in GP practice was more limited. However, a large proportion (>40%) of responding GPs indicated that many of the clinical practice survey questions were not applicable to their practice, which may suggest that these GPs do not routinely prescribe isotretinoin. Among community pharmacists, overall implementation of the PPP in practice was good, although this was predominantly focused on new patients, suggesting the need for improvement to ensure that the PPP is implemented at each dispensing. There was also wide variability in the use of educational materials in practice across the HCP groups. It should, however, be noted that the patient reminder card must now be included as part of the packaging of all oral isotretinoin products.

Overall, these findings suggest that awareness amongst specialist prescribers of the teratogenic risks of isotretinoin and implementation of all aspects of the PPP was high. While awareness on the detail of the nature of the risks was lower amongst GPs and pharmacists and some variability in execution of certain elements of the PPP was evident, the vast majority of HCPs were aware of the correct action to take in the event of an unplanned pregnancy. Amongst all HCPs, reinforcement of the provision and utilisation of the patient reminder card to support patient counselling at the level of prescription and at each dispensing may be of value. In addition, further targeted education and training amongst HCPs to complement existing risk minimisation materials may be beneficial in order to support the HCPs' role implementing the revised PPP into clinical practice.

### 4.2 Comparison with previous studies

To our knowledge, we are not aware of prior published studies which have assessed HCP's awareness, knowledge, and implementation of the 2018‐revised isotretinoin PPP in practice. Previous studies which have examined the implementation of and adherence to earlier isotretinoin PPPs, have largely revealed poor HCP uptake and compliance in practice.^7^, ^9^, ^10^, ^11^ A 2011 systematic review of available studies of compliance with the PPP for isotretinoin in Europe, that included two HCP survey studies, found insufficient compliance (6%–26%) and that the PPP needed to be strengthened.^7^ A prior survey study among dermatologists showed a high level of information (both verbal and written) provided to women around pregnancy prevention. However, the study also found that the surveyed HCP group was not consistently adherent to all aspects of risk minimisation, including exclusion of pregnancy prior to treatment.^18^ In a further study among different HCP groups in Belgium, low awareness of the isotretinoin PPP, and low compliance with some recommendations, was found; though most responding HCPs did inform WCBP on the teratogenic risks and importance of effective contraception,^19^ similar to the present study. In Ireland, a recent study examined isotretinoin prescribing practices among GPs, and found that all GPs reporting to prescribe isotretinoin for women of childbearing age (*n* = 43) also stated that they prescribed contraception when it was for women of childbearing age.^20^ The study also found that 79% (*n* = 34/43) of those GPs conducted monthly pregnancy tests, though it does not report on the proportion of GPs who conducted a pregnancy test before starting treatment with isotretinoin. Excluding GP, respondents who responded “not applicable”, these findings are broadly similar to the present study. While we are unaware of any previous studies which have examined the compliance of HCPs in Ireland with all elements of earlier isotretinoin PPPs, the levels of awareness, knowledge and implementation of the revised PPP in practice, which we report among the HCPs examined in this present survey study, suggests an improvement when compared with previous European studies examining earlier isotretinoin PPPs.^7^, ^9^, ^10^, ^11^

### 4.3 Strengths and limitations

This study incorporated the perspectives of all relevant HCPs including GPs, community pharmacists, and specialists. The HCP respondents (GPs and community pharmacists) were demographically (by age) representative of their national HCP population in Ireland. The study questionnaire developed in collaboration with the Health Products Regulatory Authority, allowed for a novel insight into the implementation of the revised isotretinoin PPP in current clinical practice in Ireland. Limitations that must be acknowledged include, the low response rates, which may limit generalisability of the survey findings. Specifically, although survey respondents were demographically similar, non‐response bias may have under‐ or over‐estimated HCPs' awareness, knowledge, and/or experience of the revised PPP for isotretinoin, which should be considered when interpreting the survey results. A recently published Irish survey study reports similar HCP response rates,^21^ which suggests it is difficult to recruit these HCPs for survey studies. Further, given the relationship between the dissemination of the DHPC and related educational materials to HCPs, as well as the significance/novelty of changes made to the PPP, and the resulting awareness and implementation of these changes in clinical practice, as the survey was conducted 6–8 months after dissemination of the full conditions and educational materials of the revised 2018 PPP to the intended HCPs, it is possible that insufficient time had elapsed to permit full implementation of the revised PPP in practice. Although anonymous online surveys were used, the possibility for desirability bias should be considered when interpreting our findings. It is also possible that respondents and non‐respondents differed in characteristics that were not captured, which may have resulted in a non‐representative sample. Therefore, it is important to consider non‐response bias and other biases associated with missing data when interpreting the survey results. In addition, area of specialisation was not surveyed for specialists, and therefore we were unable to differentiate dermatologists' responses from those of obstetricians/gynecologists. Also, responses from community pharmacists are limited to those who completed the survey, as information on “area of practice” was only collected at the end of the survey. The questionnaires did not include a screening question or skip‐pattern to mitigate the inclusion of respondents for whom questions were not applicable; however, we did seek to address this when presenting the results (e.g., through the use of contingent questions and subgroup analyses). Further, while a large proportion of GP respondents indicated that several clinical practice survey questions were not applicable, we did not survey reasons for why this may have been the case. However, our results showed that approximately half of responding GPs never prescribe isotretinoin and that the majority had not initiated a new prescription for isotretinoin in the past 12 months, which is similar to the findings of a recent study that examined isotretinoin‐prescribing practices among GPs in Ireland.^20^

### 4.4 Implications of findings

In general, although awareness, knowledge, and implementation of key elements of the revised PPP in practice was good to high (30.4% to 100%) for the specialists responding to this survey study, the findings of this study highlight that continued education and periodic reminders may be of value for all HCPs. In particular, continued supports for GPs and community pharmacists may be required, that complement currently available risk minimisation materials in order to support knowledge confidence, risk communication with patients, and reinforcement of the HCPs role in clinical practice. At clinical practice level, implementation could be verified and routinely monitored through additional measures including clinical audit, to aid compliance and identify gaps, barriers, and opportunities for continuous improvement. In addition, technology and digital tools, including clinical decision support systems, could also support cohesive and coordinated implementation of the PPP nationally, facilitating improved monitoring of the implementation in practice. However, further research is needed to fully understand the barriers and facilitators for why some HCPs may still have limited awareness/knowledge of the revised PPP, and why some HCPs may not be fully implementing all elements of the revised PPP in practice. An additional key component is the need for enhanced patient knowledge around their medications and importance of knowledge regarding risks and prevention of pregnancy. Further studies are needed among WCBP prescribed isotretinoin to examine their awareness and knowledge of the PPP.

## 5 CONCLUSION

In conclusion, we observed some variability in the level of awareness, knowledge, and implementation in clinical practice of the revised PPP for isotretinoin across the HCP groups examined. Further targeted education and/or supports on some key elements of the PPP may be of value for GPs and community pharmacists. Amongst all HCPs, reinforcement of the provision and utilisation of the patient reminder card to support patient counselling at the level of prescription and at each dispensing may also be beneficial. Additionally, further research in respect of GP practice and compliance with the revised PPP is warranted to improve understanding of current practice in Ireland.

## AUTHOR CONTRIBUTIONS

Kathleen E. Bennett, Maeve Mullooly, Niamh Buckley, Yvonne Looney (and Almath Spooner) were involved in the concept and design of the study. Survey design, data collection, and analysis were undertaken by Kathleen E. Bennett, John E. Hughes, Maeve Mullooly, Niamh Buckley, Yvonne Looney, and Gráinne Kirwan. All authors were involved in the interpretation of the data. John E. Hughes, Maeve Mullooly, and Kathleen E. Bennett prepared the manuscript. Niamh Buckley, Yvonne Looney, and Gráinne Kirwan reviewed all drafts of the manuscript and provided feedback. All authors read and approved the final manuscript.

## FUNDING INFORMATION

This study was supported by the Health Research Board Applied Partnership Award (APA‐17‐015) and Research Leader Award (RL‐15‐1579). Maeve Mullooly is supported by funding from the Health Research Board (Emerging Investigator Award EIA‐2019‐012).

## CONFLICT OF INTEREST

All authors declare that they have no conflicts of interest.

## ETHICS STATEMENT

The study received ethical approval from the Royal College of Surgeons in Ireland (RCSI) research ethics committee on 5th June 2019.

## Supporting information


**Appendix S1** Supporting Information.

**Table S1** Demographics of participating HCP groups (n [%])
**Table S2** Table showing the representativeness of Pharmacist survey responders compared to the general HCP population
**Table S3** Table showing the representativeness of General Practitioner (GP) survey responders compared to the general HCP population
**Table S4** Survey question 2, Source of information in relation to the revised PPP, required for oral Isotretinoin (Roaccutane®) in 2018/2019
**Table S5** Subgroup analysis to examine awareness/knowledge/experience among the responding GPs and specialists who indicated that they (i) frequently/occasionally prescribe, or (ii) never prescribe Roaccutane®.

## Data Availability

The data that support the findings of this study are available from the corresponding author upon reasonable request. The data are not publicly available due to privacy or ethical restrictions.
