# Supplementary material for: Evaluating awareness, knowledge and practice of healthcare professionals following implementation of a revised pregnancy prevention programme for isotretinoin in Ireland: A multi‐stakeholder cross‐sectional study
Source: Pharmacoepidemiol Drug Saf. 2022 Oct 5;32(2):137–47. doi: 10.1002/pds.5538 (PMC10092126; doi:10.1002/pds.5538)
Supplement: Supplementary file 1 — Appendix S1 Supporting Information. [file PDS-32-137-s001.pdf]

Electronic Supplementary Material 1

Article Title: Evaluating awareness, knowledge and practice of healthcare professionals following implementation of a revised Pregnancy Prevention Programme for isotretinoin in Ireland: a multi-stakeholder cross-sectional study.

Journal name: Pharmacoepidemiology & Drug Safety

Authors: John E Hughes[1], Niamh Buckley[3], Yvonne Looney[3], Gráinne Kirwan[3], Maeve Mullooly[1]†, Kathleen E. Bennett[1,2]†*

Affiliations and institutions:
[1]School of Population Health, RCSI University of Medicine and Health Sciences, Dublin 2, Ireland.
[2]Data Science Centre, School of Population Health, RCSI University of Medicine and Health Sciences, Dublin 2, Ireland.
[3]Health Products Regulatory Authority, Earlsfort Terrace, Dublin 2, Ireland.

†Maeve Mullooly and Kathleen E. Bennett are joint senior authors.

***Corresponding author:**
Kathleen E. Bennett, Data Science Centre, School of Population Health, RCSI University of Medicine and Health Sciences, Dublin 2, Ireland; Email: kathleenebennett@rcsi.ie

Survey for General Practitioners

Pregnancy Prevention Programme for oral isotretinoin (Roaccutane): Survey for GPs 2019

Welcome to the Pregnancy Prevention Programme for oral isotretinoin (Roaccutane) Survey for GPs

Thank you for your interest in this survey, please confirm the following statements:

I confirm that I have read the introductory information, email information and email invitation for this survey.

I understand that by completing this anonymous questionnaire, I consent to my data being processed and analysed as part of this survey and included in any report/publications as a result of the findings.

I understand that my data will be retained on a secure server and for a short period will be held until the research is completed and it will then be deleted.

Please click next to confirm that you have read, understand and consent to the above.

Welcome to the Pregnancy Prevention Programme for oral isotretinoin (Roaccutane) Survey for GPs

Thank you for participating in our survey, your feedback on the Pregnancy Prevention Programme is critical to evaluate its effectiveness in clinical practice. The survey consists of four sections (A-D) and it should not take more than 10 minutes to complete. We ask that you answer the questions as candidly as possible in order for the results to be of value in facilitating the optimisation and further development of the Pregnancy Prevention Programmes in Ireland.

A: Health care professional awareness

* 1. Did you receive any information in relation to the revised Pregnancy Prevention Programme, required for oral isotretinoin (Roaccutane) in 2018/2019?

- ○ Yes
- ○ No

2. What was the source (s) of this information?*(you may select more than one option, if applicable)?*

- [ ] Dear Doctor letter (from HPRA & pharmaceutical company)
- [ ] Drug Safety Newsletter from HPRA
- [ ] Educational materials (e.g. Physician Checklist)
- [ ] Continuing professional development activity
- [ ] Work colleague(s)
- [ ] Professional society
- [ ] Patient(s) prescribed oral isotretinoin

Other (please specify)

* 3. Please indicate which statement in the following pairs is most accurate, a or b:

	(a) or (b) or not sure
a) Oral isotretinoin is contraindicated in any woman of childbearing potential<br><br>b) Oral isotretinoin is contraindicated in any woman of childbearing potential unless the conditions of the pregnancy prevention programme are fulfilled	
a) Exposure to oral isotretinoin during pregnancy can cause severe foetal malformations and spontaneous abortion<br><br>b) Exposure to oral isotretinoin during pregnancy can cause severe foetal malformations	

	(a) or (b) or not sure
a) Use of oral isotretinoin, at therapeutic dosages, by male patients has not been associated with foetal malformations<br><br>b) Use of oral isotretinoin, at therapeutic dosages, by male patients has been associated with foetal malformations	
a) Female patients should be advised to continue to use effective contraception for at least one month after stopping treatment with oral isotretinoin<br><br>b) Female patients should be advised to continue to use effective contraception for at least two months after stopping treatment with oral isotretinoin	

* 4. Are you aware of the existence of the following educational materials to support the implementation of the Pregnancy Prevention Programme for Roaccutane?

	Yes	No
Physician checklist	◯	◯
Pharmacist checklist	◯	◯
Patient reminder card	◯	◯

* 5. Have you used any of the relevant aforementioned educational materials for Roaccuatane when implementing the Pregnancy Prevention Programme in your clinical practice? (please indicate any/all that you have used)?

	Yes	No	Not applicable
Physician checklist	◯	◯	◯
Patient reminder card	◯	◯	◯

Please provide a brief explanation if 'Not applicable' is ticked

* 6. Do you have hard copies of these educational materials, provided to you by the company, currently available in your practice?

	Yes	No	Not applicable
Physician checklist	◯	◯	◯
Patient reminder card	◯	◯	◯

Please provide a brief explanation if 'No' or ‘Not applicable’ is ticked

B: Prescribing Practice

* 7. When was the last time you initiated a **new** prescription for Roaccutane?

- In the past week
- In the past month
- In the past 3 months
- In the past 6 months
- In the past 12 months
- Did not initiate a new prescription for Roaccutane in the past 12 months

* 8. When was the last time you **renewed** a prescription for Roaccutane?

- In the past week
- In the past month
- In the past 3 months
- In the past 6 months
- In the past 12 months
- Did not renew a prescription for Roaccutane in the past 12 months

* 9. On average, how frequently do you prescribe Roaccutane?

- Frequently (greater than once per week)
- Occasionally (about once per month)
- Rarely (about once every 3-6 months)
- Very rarely (about once every 12 months)
- Never

C. Implementation of the Pregnancy Prevention Program for Roaccutane into your clinical practice

* 10. Which, if any, of the following have you implemented in your clinical practice with women of child-bearing potential when **initiating Roaccutane**? (Please complete the table below)

	Yes	No	Not applicable
I considered alternative treatment options before initiating my patient on Roaccutane	◯	◯	◯
I initiated my patient on effective contraception (if not already using contraception)	◯	◯	◯
I ensured my patient understood the need to comply with contraception for 1 month before starting treatment, throughout treatment and for 1 month after the end of treatment	◯	◯	◯
I requested my patient to have a pregnancy test before starting treatment with Roaccutane	◯	◯	◯
I ensured my patient understood the teratogenic risks associated with the use of Roaccutane in pregnancy	◯	◯	◯
I ensured my patient had a copy of the patient reminder card	◯	◯	◯

Other (please specify)

* 11. Which, if any, of the following have you implemented in your clinical practice with women of child-bearing potential when **renewing a prescription for Roaccutane**? (Please complete the table below)

	Yes	No	Not applicable
I limit my prescriptions to 30 days to support regular follow-up	○	○	○
When indicated I request my patient to have a pregnancy test	○	○	○

* 12. In the event of an **unplanned or suspected pregnancy** in your patient currently being prescribed Roaccutane, which, if any, of the following would you implement in your clinical practice?

	Yes	No	Not applicable
I would advise my patient to consult with me urgently	○	○	○
I would advise my patient to stop taking Roaccutane immediately	○	○	○
I would reduce my patient's Roaccutane dose	○	○	○

Other (please specify)

* 13. At what stage(s) in the patient journey do you review the teratogenic risks of Roaccutane with female patients? (please tick all that apply)

- ☐ On initiation of Roaccutane
- ☐ Patient is planning a pregnancy
- ☐ Patient is pregnant
- ☐ Annually
- ☐ At every consultation
- ☐ Do not review teratogenic risks with patients
- ☐ Not applicable

Other (please specify)

* 14. Have there been any clinical scenarios in your practice where you adapted the Pregnancy Prevention Programme for Roaccutane to an individual patient’s circumstances?

- ◯ Yes
- ◯ No
- ◯ Not applicable

If yes, please describe the scenario(s)

15. Please provide any further comments/observations on the Pregnancy Prevention Programme for Roaccutane below.

D: Demographic Information

Finally, we would like to ask a few questions about you.

16. Please indicate your age range:

- ◯ 25-34 years
- ◯ 35-44 years
- ◯ 45-54 years
- ◯ 55-64 years
- ◯ >64 years
- ◯ Prefer not to say

17. Please indicate your gender:

- ◯ Male
- ◯ Female
- ◯ Prefer not to say
- ◯ Other

18. Please indicate the number of years you have been in GP practice:

- ◯ < 5 years
- ◯ 5 – 10 years
- ◯ 11 – 15 years
- ◯ 16 – 20 years
- ◯ 21 – 25 years
- ◯ > 25 years
- ◯ Prefer not to say

19. Please indicate the number years since you qualified as a doctor (since completion of undergraduate medical training):

- ◯ < 5 years
- ◯ 5 – 10 years
- ◯ 11 – 15 years
- ◯ 16 – 20 years
- ◯ 21 - 25 years
- ◯ > 25 years
- ◯ Prefer not to say

Thank you for taking the time to complete this survey.

Survey for Pharmacists

Pregnancy Prevention Programme for oral isotretinoin (Roaccutane®): Survey for Pharmacists 2019

Welcome to the Pregnancy Prevention Programme for oral isotretinoin (Roaccutane®) Survey

Thank you for your interest in this survey, please confirm the following statements:

I confirm that I have read the introductory information, email information and email invitation for this survey.

I understand that by completing this anonymous questionnaire, I consent to my data being processed and analysed as part of this survey and included in any report/publications as a result of the findings.

I understand that my data will be retained on a secure server and for a short period will be held until the research is completed and it will then be deleted.

Please click next to confirm that you have read, understand and consent to the above.

Welcome to the Pregnancy Prevention Programme for oral isotretinoin (Roaccutane®) Survey

Thank you for participating in our survey, your feedback on the Pregnancy Prevention Programme is critical to evaluate its effectiveness in clinical practice. The survey consists of three sections (A-C) and it should not take more than 10 minutes to complete. We ask that you answer the questions as candidly as possible in order for the results to be of value in facilitating the optimisation and further development of Pregnancy Prevention Programmes in Ireland.

A: Healthcare professional awareness

* 1. Did you receive any information in relation to the revised Pregnancy Prevention Programme, required for oral isotretinoin (Roaccutane®) in 2018/2019?

- ◯ Yes
- ◯ No
- ◯ Can't remember

A: Healthcare professional awareness

* 2. What was the source(s) of this information?*(you may select more than one option, if applicable)*

- ☐ Dear Pharmacist letter (from HPRA & pharmaceutical company)
- ☐ Drug Safety Newsletter from HPRA
- ☐ Educational materials (e.g. Pharmacist Checklist)
- ☐ Continuing professional development activity
- ☐ Work colleague(s)
- ☐ Professional society
- ☐ Patient(s) prescribed oral isotretinoin
- ☐ Other

If other, please specify

A: Healthcare professional awareness

* 3. Please indicate which statement in the following pairs is most accurate, a or b:

	(a) or (b) or not sure
a) Oral isotretinoin is contraindicated in any woman of childbearing potential<br><br>b) Oral isotretinoin is contraindicated in any woman of childbearing potential unless the conditions of the pregnancy prevention programme are fulfilled	
a) Exposure to oral isotretinoin during pregnancy can cause severe foetal malformations and spontaneous abortion<br><br>b) Exposure to oral isotretinoin during pregnancy can cause severe foetal malformations	
a) Use of oral isotretinoin, at therapeutic dosages, by male patients has not been associated with foetal malformations<br><br>b) Use of oral isotretinoin, at therapeutic dosages, by male patients has been associated with foetal malformations	
a) Female patients should be advised to continue to use effective contraception for at least one month after stopping treatment with oral isotretinoin<br><br>b) Female patients should be advised to continue to use effective contraception for at least two months after stopping treatment with oral isotretinoin	

* 4. Are you aware of the existence of the following educational materials to support the implementation of the Pregnancy Prevention Programme for Roaccutane®?

	Yes	No
Physician checklist	◯	◯
Pharmacist checklist	◯	◯
Patient reminder card	◯	◯

* 5. Have you used any of the relevant aforementioned educational materials for Roaccutane® when implementing the Pregnancy Prevention Programme in your pharmacy? (*please indicate any/all that you have used*)

	Yes	No	Not applicable
Pharmacist checklist	◯	◯	◯
Patient reminder card	◯	◯	◯

Please provide a brief explanation if 'Not applicable' is ticked

* 6. Do you have hard copies of these educational materials, provided to you by the company, currently available in your pharmacy?

	Yes	No	Not applicable
Pharmacist checklist	◯	◯	◯
Patient reminder card	◯	◯	◯

Please provide a brief explanation if 'Not applicable' is ticked

B. Implementation of the Pregnancy Prevention Programme

* 7. Have you dispensed Roaccutane® in the past 12 months?

- ◯ Yes
- ◯ No
- ◯ Not applicable

Please provide a brief explanation if 'Not applicable' is ticked

* 8. When dispensing Roaccutane®, which, if any, of the following have you implemented in your pharmacy practice with **women of child-bearing potential** currently being prescribed Roaccutane®? (*Please complete the table below*)

	Yes	No	Not applicable
I ensured Roaccutane® was only dispensed within a maximum of 7 days of the prescription	◯	◯	◯
Where possible, I ensured the prescription was limited to a 30 day supply	◯	◯	◯
I referred my patient to her GP as she was not using effective contraception	◯	◯	◯

* 9. When dispensing Roaccutane®, which, if any, of the following have you implemented in your pharmacy practice with **men and women of child-bearing potential** currently being prescribed Roaccutane®?

	Yes for men	Yes for women of child-bearing potential	Not applicable
I advised my patient never to share their Roaccutane® with another person	☐	☐	☐
I advised my patient to return any unused capsules to me at the end of the month	☐	☐	☐
I advised my patient not to donate blood during Roaccutane® therapy and for one month after discontinuation	☐	☐	☐

* 10. Please answer the following questions in relation to your pharmacy practice when dispensing Roaccutane® to **women of child-bearing potential**:

	Only for new patients	At each dispensing	Only if patient initiates discussion	Never	Not applicable
How often do you provide the patient card to your patient and counsel her on its contents	○	○	○	○	○
How often do you counsel the patient on the teratogenic risks associated with use of Roaccutane® in pregnancy	○	○	○	○	○
How often do you reinforce the need for effective contraception	○	○	○	○	○

* 11. When dispensing Roaccutane® to a **male patient**, how often do you provide the patient reminder card and counsel him on the relevant aspects of its contents?

○ Only for new patients

○ At each dispensing

○ Only if patient initiates discussion

○ Never

○ Not applicable

* 12. When dispensing Roaccutane® and broken bulk dispensing cannot be avoided, which, if any, of the following do you provide to the patient (*please tick all that apply*):

☐ Roaccutane® package leaflet/patient information leaflet (only for women of childbearing potential)

☐ Roaccutane® package leaflet/patient information leaflet (for men and women of childbearing potential)

☐ Roaccutane® patient card (only for women of childbearing potential)

☐ Roaccutane® patient card (for men and women of childbearing potential)

☐ None of the above.

☐ Not applicable

* 13. In the case of an **unplanned or suspected pregnancy** in a woman currently being prescribed Roaccutane® which, if any, of the following actions would you take?

	Yes	No
I would advise my patient to contact her prescribing doctor immediately	○	○
I would advise my patient to stop taking Roaccutane® immediately	○	○
I would not dispense Roaccutane® to my patient	○	○
I would counsel my patient on the teratogenic risks associated with use of Roaccutane® in pregnancy	○	○

Other (please specify)

14. Please provide any further comments/observations on the Pregnancy Prevention Programme for Roaccutane® below.

C: Demographic Information

Finally, we would like to ask a few questions about you.

15. Please indicate your age range:

- ◯ 22-34 years
- ◯ 35-44 years
- ◯ 45-54 years
- ◯ 55-64 years
- ◯ >64 years
- ◯ Prefer not to say

16. Please indicate your gender:

- ◯ Male
- ◯ Female
- ◯ Prefer not to say
- ◯ Other

17. Please indicate your primary area of practice:

- ◯ Community pharmacy
- ◯ Hospital pharmacy
- ◯ Academia
- ◯ Other (please specify)
- ◯ Industry
- ◯ Regulatory
- ◯ Prefer not to say

18. Please indicate the number years since you qualified as a pharmacist:

- ◯ < 5 years
- ◯ 5 – 10 years
- ◯ 11 – 15 years
- ◯ 16 – 20 years
- ◯ 21 - 25 years
- ◯ > 25 years
- ◯ Prefer not to say

Thank you for taking the time to complete this survey.

Survey for Specialists

Pregnancy Prevention Programme for oral isotretinoin (Roaccutane): Survey for Specialists 2019

Welcome to the Pregnancy Prevention Programme for oral isotretinoin (Roaccutane) Survey

Thank you for your interest in this survey, please confirm the following statements:

I confirm that I have read the introductory information, email information and email invitation for this survey.

I understand that by completing this anonymous questionnaire, I consent to my data being processed and analysed as part of this survey and included in any report/publications as a result of the findings.

I understand that my data will be retained on a secure server and for a short period will be held until the research is completed and it will then be deleted.

Please click next to confirm that you have read, understand and consent to the above.

Welcome to the Pregnancy Prevention Programme for oral isotretinoin (Roaccutane) Survey

Thank you for participating in our survey. Your feedback on the Pregnancy Prevention Programme is critical to evaluate its effectiveness in clinical practice. The survey consists of four sections (A-D) and it should not take more than 10 minutes to complete. We ask that you answer the questions as candidly as possible in order for the results to be of value in facilitating the optimisation and further development of Pregnancy Prevention Programmes in Ireland.

A: Healthcare professional awareness

* 1. Did you receive any information in relation to the revised Pregnancy Prevention Programme, required for oral isotretinoin (Roaccutane) in 2018/2019?

- ◯ Yes
- ◯ No

A: Healthcare professional awareness

* 2. What was the source (s) of this information?*(you may select more than one option, if applicable)*

- [ ] Dear Doctor letter (from HPRA & pharmaceutical company)
- [ ] Drug Safety Newsletter from HPRA
- [ ] Educational materials (e.g. Physician Checklist)
- [ ] Continuing professional development activity
- [ ] Work colleague(s)
- [ ] Professional society
- [ ] Patient(s) prescribed oral isotretinoin
- [ ] Other

If other (please specify)

A: Healthcare professional awareness

* 3. Please indicate which statement in the following pairs is most accurate, a or b:

	(a) or (b) or not sure
a) Oral isotretinoin is contraindicated in any woman of childbearing potential<br><br>b) Oral isotretinoin is contraindicated in any woman of childbearing potential unless the conditions of the pregnancy prevention programme are fulfilled	
a) Exposure to oral isotretinoin during pregnancy has been associated with severe foetal malformations and spontaneous abortion<br><br>b) Exposure to oral isotretinoin during pregnancy has been associated with severe foetal malformations	
a) Use of oral isotretinoin, at therapeutic dosages, by male patients has not been associated with foetal malformations<br><br>b) Use of oral isotretinoin, at therapeutic dosages, by male patients has been associated with foetal malformations	
a) Female patients should be advised to continue to use effective contraception for at least one month after stopping treatment with oral isotretinoin<br><br>b) Female patients should be advised to continue to use effective contraception for at least two months after stopping treatment with oral isotretinoin	

* 4. Are you aware of the existence of the following educational materials to support the implementation of the Pregnancy Prevention Programme for Roaccutane?

	Yes	No
Physician checklist	○	○
Pharmacist checklist	○	○
Patient reminder card	○	○

* 5. Have you used any of the relevant aforementioned educational materials for Roaccuatane when implementing the Pregnancy Prevention Programme in your clinical practice? (please indicate any/all that you have used)

	Yes	No	Not applicable
Physician checklist	○	○	○
Patient reminder card	○	○	○

Please provide a brief explanation if ‘Not applicable’ is ticked

* 6. Do you have hard copies of these educational materials, provided to you by the company, currently available in your practice?

	Yes	No	Not applicable
Physician checklist	○	○	○
Patient reminder card	○	○	○

Please provide a brief explanation if 'No' or ‘Not applicable’ is ticked

B: Prescribing Practice

* 7. When was the last time you initiated a **new** prescription for Roaccutane?

- In the past week
- In the past month
- In the past 3 months
- In the past 6 months
- In the past 12 months
- Did not initiate a new prescription for Roaccutane in the past 12 months

* 8. When was the last time you **renewed** a prescription for Roaccutane?

- In the past week
- In the past month
- In the past 3 months
- In the past 6 months
- In the past 12 months
- Did not renew a prescription for Roaccutane in the past 12 months

* 9. On average, how frequently do you prescribe Roaccutane?

- Frequently (greater than once per week)
- Occasionally (about once per month)
- Rarely (about once every 3-6 months)
- Very rarely (about once every 12 months)
- Never

C. Implementation of the Pregnancy Prevention Programme for Roaccutane into your clinical practice

* 10. Which, if any, of the following have you implemented in your clinical practice with women of child-bearing potential when **initiating Roaccutane**? (Please complete the table below)

	Yes	No	Not applicable
I considered alternative treatment options before initiating my patient on Roaccutane	○	○	○
I initiated my patient on effective contraception (if not already using contraception)	○	○	○
I referred my patient to her GP/family planning clinic for contraceptive advice (if not already using contraception)	○	○	○
I ensured my patient understood the need to comply with contraception for 1 month before starting treatment, throughout treatment and for 1 month after the end of treatment	○	○	○
I requested my patient to have a pregnancy test before starting treatment with Roaccutane	○	○	○
I ensured my patient understood the teratogenic risks associated with use of Roaccutane in pregnancy	○	○	○
I ensured my patient had a copy of the patient reminder card	○	○	○

Other (please specify)

* 11. In the event of an **unplanned or suspected pregnancy** in your patient currently being prescribed Roaccutane, which, if any, of the following would you implement in your clinical practice?

	Yes	No
I would advise my patient to consult with me urgently	◯	◯
I would advise my patient to consult with her GP urgently	◯	◯
I would advise my patient to stop taking Roaccutane immediately	◯	◯
I would reduce my patient's Roaccutane dose	◯	◯

Other (please specify)

* 12. At what stage(s) in the patient journey do you review the teratogenic risks of Roaccutane with female patients? (please tick all that apply)

- ☐ On initiation of Roaccutane
- ☐ If patient is planning a pregnancy
- ☐ Patient is pregnant
- ☐ Annually
- ☐ At every consultation
- ☐ Do not review teratogenic risks with patients
- ☐ Not applicable

Other (please specify)

* 13. Have there been any clinical scenarios in your practice where you adapted the Pregnancy Prevention Programme for Roaccutane to an individual patient's circumstances?

- ◯ Yes
- ◯ No
- ◯ Not applicable

If yes, please describe the scenario(s)

14. Please provide any further comments/observations on the Pregnancy Prevention Programme for Roaccutane below.

D: Demographic Information

Finally, we would like to ask a few questions about you.

15. Please indicate your age range:

- ○ 25-34 years
- ○ 35-44 years
- ○ 45-54 years
- ○ 55-64 years
- ○ >64 years
- ○ Prefer not to say

16. Please indicate your gender:

- ○ Male
- ○ Female
- ○ Other
- ○ Prefer not to say

17. Please indicate the number of years you have been practising as a specialist (since completion of higher specialist training):

- ○ < 5 years
- ○ 5 – 10 years
- ○ 11 – 15 years
- ○ 16 – 20 years
- ○ 21 – 25 years
- ○ > 25 years
- ○ Prefer not to say

18. Please indicate the number years since you qualified as a doctor (since completion of undergraduate medical training):

- ○ < 5 years
- ○ 5 – 10 years
- ○ 11 – 15 years
- ○ 16 – 20 years
- ○ 21 – 25 years
- ○ > 25 years
- ○ Prefer not to say

Thank you for taking the time to complete this survey.
