# Supplementary material for: Evaluating awareness, knowledge and practice of healthcare professionals following implementation of a revised pregnancy prevention programme for isotretinoin in Ireland: A multi‐stakeholder cross‐sectional study
Source: Pharmacoepidemiol Drug Saf. 2022 Oct 5;32(2):137–47. doi: 10.1002/pds.5538 (PMC10092126; doi:10.1002/pds.5538)
Supplement: Supplementary file 2 — Table S1 Demographics of participating HCP groups (n [%]) Table S2 Table showing the representativeness of Pharmacist survey responders compared to the general HCP population Table S3 Table showing the representativeness of General Practitioner (GP) survey responders compared to the general HCP population Table S4 Survey question 2, Source of information in relation to the revised PPP, required for oral Isotretinoin (Roaccutane®) in 2018/2019 Table S5 Subgroup analysis to examine awareness/knowledge/experience among the responding GPs and specialists who indicated that they (i) frequently/occasionally prescribe, or (ii) never prescribe Roaccutane®. [file PDS-32-137-s002.docx]

**Electronic Supplementary Material 2**

**Article Title**: Evaluating awareness, knowledge and practice of healthcare professionals following implementation of a revised Pregnancy Prevention Programme for isotretinoin in Ireland: a multi-stakeholder cross-sectional study.

**Journal name:** Pharmacoepidemiology & Drug Safety

**Authors:** John E. Hughes^1^, Niamh Buckley^3^, Yvonne Looney^3^, Gráinne Kirwan^3^, Maeve Mullooly^1^†, Kathleen E. Bennett^1,2^†*

**Affiliations and institutions:**

^1^School of Population Health, RCSI University of Medicine and Health Sciences, Dublin 2, Ireland.

^2^Data Science Centre, School of Population Health, RCSI University of Medicine and Health Sciences, Dublin 2, Ireland.

^3^Health Products Regulatory Authority, Earlsfort Terrace, Dublin 2, Ireland.

†Maeve Mullooly and Kathleen E. Bennett are joint senior authors.

***Corresponding author:**

Kathleen E. Bennett, Data Science Centre, School of Population Health, RCSI University of Medicine and Health Sciences, Dublin 2, Ireland; Email: [kathleenebennett@rcsi.ie](mailto:kathleenebennett@rcsi.ie)

**Table S1: Demographics of participating HCP groups (n (%))**

|  |  | **GPs^a, b^**  **(N=75)** | **Community Pharmacists^a^ (N=111)** | **Specialists^a, b^**  **(N=21)** |
| --- | --- | --- | --- | --- |
| **Age groups** | 25-34 years | 9 (12%) | 42 (37.8%) ^#^ | 5 (23.8%) |
|  | ≥35 years | 65 (86.7%) | 68 (61.3%) | 15 (71.4%) |
|  | Prefer not to say | n<5 | n<5 | n<5 |
| **Gender** § |  |  |  |  |
|  | Male | 23 (30.7%) | 34 (30.6%) | n<5 |
|  | Female | 50 (66.7%) | 76 (68.5%) | 17 (81.0%) |
| **Years in practice** § |  |  |  |  |
|  | < 5 years | 14 (18.7%) | - | 7 (33.3%) |
|  | 5-15 years | 26 (34.7%) | - | 5 (23.8%) |
|  | ≥16 years | 34 (45.3%) | - | 7 (33.3%) |
| **Years since qualification** § |  |  |  |  |
|  | <5 years | 0 (0.0%) | 20 (18.0%) | 0 (0.0%) |
|  | 5-15 years | 25 (33.3%) | 44 (39.6%) | 6 (28.6%) |
|  | 16-25 years | 25 (33.3%) | 27 (24.3%) | 7 (33.3%) |
|  | > 25 years | 24 (32.0%) | 18 (16.2%) | 7 (33.3%) |

*Data presented as n (%), unless otherwise stated*.

The dashed line indicates that this question/answer was not asked/included in this HCP survey

^a^ Demographic percentages were calculated based upon number of HCPs who completed the survey to the end

^b^ 23 GPs and 5 Specialists had missing data.

^#^ Youngest age group for pharmacists was 22-34years

^§^ Where the numbers do not add to total, this is due to exclusion of n<5 (prefer not to say or no response)

**Table S2: Table showing the representativeness of Pharmacist survey responders compared to the general HCP population**

|  | **Pharmacist Survey respondents^$^**  **(N=143)** | **Pharmacist HCP Population^&^*** |
| --- | --- | --- |
| **Age range** | % | % |
| 22-34 years | 39.6% | 36.4% |
| 35-44 years | 31.7% | 32.7% |
| 45-54 years | 17.1% | 15.5% |
| 55-64 years | 9.2% | 6.8% |
| >64 years | 1.2% | 2.1% |
| Prefer not to say | 1.2% | - |
| **Gender** |  |  |
| Male | 27.4% | 35.4% |
| Female | 71.3% | 64.7% |
| Prefer not to say | 1.2% | - |
| Other | 0.0% | - |
| **Primary area of practice** |  |  |
| Community pharmacy | 67.7% | 57.5% |
| Hospital pharmacy | 17.1% | 10.2% |
| Academia | 4.3% | 1.3% |
| Industry | 3.1% | 3.3% |
| Regulatory | 2.4% | 1.4% |
| Prefer not to say | 1.8% | - |
| Other (please specify) | 3.7% | - |

^$^Survey responses collected in September 2019

^&^Pharmacist populations obtained from the PSI monthly statistics, obtained in January 2020

*Categories include 26-35 years, 36-45 years, 46-55 years, 56-65 years, >66 years

**Table S3: Table showing the representativeness of General Practitioner (GP) survey responders compared to the general HCP population**

|  | **GP Survey Respondents^§^ (N=90)** | **GP HCP Population^1^** |
| --- | --- | --- |
| **Age range** | % | % |
| 25-34 years | 12.0% | 11.6% |
| 35-44 years | 37.3% | 31.5% |
| 45-54 years | 22.7% | 24.2% |
| 55-64 years | 20.0% | 21.2% |
| >64 years | 6.7% | 11.4% |
| Prefer not to say | 1.3% | - |

^§^n=27 GPs no response

^1^Population data for 2018 obtained from ICGP (personal communication 23/01/2020)

**Table S4: Survey question 2, Source of information in relation to the revised PPP, required for oral Isotretinoin (Roaccutane®) in 2018/2019**

|  |  | **GPs (n=41)** | **Community Pharmacists (n=65)** | **Specialists (n=12)** |
| --- | --- | --- | --- | --- |
| **What was the source of this information?** ^a, b^ | Dear Dr/Pharmacist Letter | 27 (65.9%) | 53 (81.5%) | n<5 |
|  | Drug Safety Newsletter from HPRA | 13 (31.7%) | 27 (41.5%) | n<5 |
|  | Education materials | 7 (17.1%) | 29 (44.6%) | 6 (50.0%) |
|  | Other ^c^ | 6 (14.6%) | 22 (33.8%) | 6 (50.0%) |

*Data presented as n (%), unless otherwise stated*

^a^ HCP’s may select more than one option, if applicable.

^b^ As this question is contingent on Q1, it uses the number who responded “Yes” to Q1 of the survey as the denominator.

^c^ Continuing Professional Development activity, Work Colleague(s), Professional Society and Patient(s) prescribed oral Isotretinoin, and other (specified by HCP survey).

**Table S5. Subgroup analysis to examine awareness/knowledge/experience among the responding GPs and specialists who indicated that they (i) frequently/occasionally prescribe, or (ii) never prescribe Roaccutane®.**

|  | **Frequently/occasionally prescribe Roaccutane®** | | **Never prescribe Roaccutane®** | |
| --- | --- | --- | --- | --- |
|  | **GP (n=13)** | **Specialist (n=13)** | **GP (n=42)** | **Specialist (n=10)** |
| **AWARENESS/KNOWLEDGE** |  |  |  |  |
| **Did receive information relating to the revised isotretinoin PPP** | 6 (46.2%) | 9 (69.2%) | 15 (35.7%) | ~ |
| **Knowledge that “Oral isotretinoin is contraindicated in any woman of childbearing potential unless the conditions of the pregnancy prevention programme are fulfilled”** | 11 (84.6%) | 13 (100.0%) | 39 (92.6%) | 7 (70.0%) |
| **Knowledge that “Exposure to oral isotretinoin during pregnancy has been associated with severe foetal malformations and spontaneous abortion”** | 7 (53.8%) | 10 (76.9%) | 30 (71.4%) | 6 (60.0%) |
| **Knowledge that “Use of oral isotretinoin, at therapeutic dosages, by male patients has not been associated with foetal malformations”** | 10 (76.9%) | 11 (84.6%) | 10 (23.8%) | 7 (70.0%) |
| **Knowledge that “Female patients should be advised to continue to use effective contraception for at least one month after stopping treatment with oral isotretinoin”** | 5 (38.5%) | 13 (100.0%) | 5 (11.9%) | ~ |
| **Indicated awareness of the physician checklist** | 9 (69.2%) | 12 (92.3%) | 13 (31.0%) | 0 (0.0%) |
| **Indicated awareness of the patient reminder card** | 9 (69.2%) | 8 (61.5%) | 6 (14.3%) | ~ |
| **EXPERIENCE** |  |  |  |  |
| **Indicated use of the physician checklist** | 7 (53.8%) | 11 (84.6%) | ~ | 0 (0.0%) |
| **Indicated use of the patient reminder card** | 6 (46.2%) | 7 (53.8%) | ~ | 0 (0.0%) |
| **When initiating Roaccutane® in WCBP, I considered alternative treatment options before initiating my patient on Roaccutane** | 9 (69.2%) | 12 (92.3%) | 9 (21.4%) | ~ |
| **When initiating Roaccutane® in WCBP, I initiated my patient on effective contraception (if not already using contraception)** | 11 (84.6%) | 8 (61.5%) | 8 (19.0%) | ~ |
| **When initiating Roaccutane® in WCBP, I referred my patient to her GP/family planning clinic for contraceptive advice (if not already using contraception)** | *SPECIALIST SURVEY ONLY* | 12 (92.3%) | *SPECIALIST SURVEY ONLY* | ~ |
| **When initiating Roaccutane® in WCBP, I ensured my patient understood the need to comply with contraception for 1 month before starting treatment, throughout treatment and for 1 month after the end of treatment** | 11 (84.6%) | 13 (100.0%) | 10 (23.8%) | ~ |
| **When initiating Roaccutane® in WCBP, I requested my patient to have a pregnancy test before starting treatment with Roaccutane** | 9 (69.2%) | 13 (100.0%) | 5 (11.9%) | ~ |
| **When initiating Roaccutane® in WCBP, I ensured my patient understood the teratogenic risks associated with the use of Roaccutane in pregnancy** | 11 (84.6%) | 13 (100.0%) | 8 (19.0%) | ~ |
| **When initiating Roaccutane® in WCBP, I ensured my patient had a copy of the patient reminder card** | ~ | 8 (61.5%) | 0 (0.0%) | ~ |
| **When renewing a prescription for Roaccutane for WCBP, I limit my prescriptions to 30 days to support regular follow-up** | 10 (76.9%) | *GP SURVEY ONLY* | 7 (16.7%) | *GP SURVEY ONLY* |
| **When renewing a prescription for Roaccutane for WCBP, when indicated I request my patient to have a pregnancy test** | 12 (92.3%) | *GP SURVEY ONLY* | 11 (26.2%) | *GP SURVEY ONLY* |

***~ data where n<5 are not reported***
